# Ultrasensitive Optical Fiber Sensors Working at Dispersion Turning Point: Review

**DOI:** 10.3390/s23031725

**Published:** 2023-02-03

**Authors:** Shengyao Xu, Peng Kang, Zhijie Hu, Weijie Chang, Feng Huang

**Affiliations:** School of Mechanical Engineering and Automation, Fuzhou University, Fuzhou 350108, China

**Keywords:** optical fiber sensor, dispersion turning point, sensitivity enhancement, fiber grating, fiber coupler, interferometer

## Abstract

Optical fiber sensors working at the dispersion turning point (DTP) have served as promising candidates for various sensing applications due to their ultrahigh sensitivity. In this review, recently developed ultrasensitive fiber sensors at the DTP, including fiber couplers, fiber gratings, and interferometers, are comprehensively analyzed. These three schemes are outlined in terms of operation principles, device structures, and sensing applications. We focus on sensitivity enhancement and optical transducers, we evaluate each sensing scheme based on the DTP principle, and we discuss relevant challenges, aiming to provide some clues for future research.

## 1. Introduction

Optical fiber sensors with high sensitivity and compact structures have attracted tremendous interest in various applications [1,2,3]. The enormous demand for optical fiber sensors with high versatility and performance is driving the combination of micro/nanotechnology with waveguide optics [3,4]. Optical microfibers with diameters smaller than ten micrometers have engaged as promising sensing platforms due to their unique transmission characteristics. Because the diameter of such a microfiber is close to or below the wavelength of guided light, a portion of guided light escapes the microfiber in the form of evanescent fields. Moreover, due to the large refractive index difference between the fiber material and the surrounding area, the excited and highly fractional evanescent fields are tightly spatially confined. Compared with the mode field inside the fiber, the evanescent field is more susceptible to the influence of the external environment, so the sensitivity of microfibers is higher than that of standard bulk fibers. On the other hand, microfibers demonstrate excellent configurability into various microstructures, such as loops, junctions, coils, and couplers, due to their good flexibility and mechanical strength [3]. Researchers have reported various schemes of microfiber-based sensors, including micro-gratings, interferometers, micro-resonators, and surface plasmon resonance-based microfibers [5]. Although microfibers exhibit many outstanding characteristics, they have some inherent limitations, such as sensitivity and repeatability, that restrict their usefulness in sensing applications. For example, for bio-sensing, the detection limit of biomarkers depends on the sensitivity of sensors [6,7]. It is important to improve this sensitivity to meet the actual needs of the early detection and diagnosis of diseases, which is the bottleneck of traditional microfiber sensors [8]. Therefore, the sensitivity of microfibers needs to be further improved, but the mechanism between their diameter and sensing characteristics remains to be revealed. 

Recently, fiber-based sensors operating near the dispersion turning point (DTP) have attracted a high level of research interest and served as promising candidates for various sensing applications due to their ultrahigh sensitivity [9,10,11,12,13]. The DTP is the operation wavelength where the group-effective refractive index (RI) difference between the coupling modes is equal to zero [14]. In fact, the propagation constant difference of the two modes nonlinearly changes with wavelength and turns around at the DTP [15]. An ultrahigh sensitivity can be achieved by carefully modifying the fiber diameter or coating nanomaterial to mitigate the group-effective RI difference’s infinite approach towards zero, namely, the sensitivity can be significantly improved to 10^4^ nm/RIU or higher. Guided by this principle, researchers have reported on a variety of microfiber sensors operating near the DTP, such as micro-couplers [16,17], microfiber gratings [18,19], zigzag-shaped microfibers [20], interferometers [9,11,21], and micro-tips [10,13]. However, the diameter should be strictly controlled within several hundred nanometers to achieve the desired ultrahigh sensitivity, which significantly increase fabrication difficulty and degrades production repeatability. To overcome this fabrication challenges, researchers have proposed advanced flame stretching methods to control structural parameters [22,23]. A mode dispersion method to fundamentally expand the range of working diameters was reported to improve fabrication tolerance and ease manufacturing difficulties. Additionally, the high-sensitivity operation bandwidth can be significantly broadened. In this paper, we systematically review recent progress in fiber sensors based on DTP theory. Firstly, we describe the DTP sensing mechanisms and summarize typical DTP-based fiber sensors in terms of their sensing mechanisms. We also analyze the sensing performance of each type of DTP-based fiber sensor. Finally, we conclude and discuss challenges and prospects in fiber sensors based on DTP theory.

## 2. DTP Sensing Mechanisms and Recent Advances

The fundamental sensing idea for optical fibers comprises light–environment interactions. In particular, by tapering or etching a fiber, a large proportion of light can propagate along fiber in the form of evanescent fields that are sensitive to the external environment [23,24]. The environmental physical/chemical parameters can be demodulated by detecting the transmitted or reflected spectrum. Furthermore, by carefully controlling the structural parameters of the fiber, such as by decreasing its diameter and etching cladding, the transmission characteristics can be flexibly tailored and engineered. In this way, the group reflective index difference between coupling modes can be adjusted to approach zero, which is defined as the DTP, so the sensitivity can be infinitely improved. Recent years have witnessed rapid developments in DTP-based fiber sensors, and numerous works have been published. In this section, we catalogue them into three main types according to sensing mechanism: fiber coupler, fiber grating, and in-fiber interferometer (although crossovers may occur).

### 2.1. Fiber Coupler

A fiber coupler is an essential device of many optic fiber setups. Two or more fibers are tapered to the micron size so that the fiber cores intimate contact each other, allowing light from one or several input fibers to couple to one or several output fibers. 

**Figure 1:**
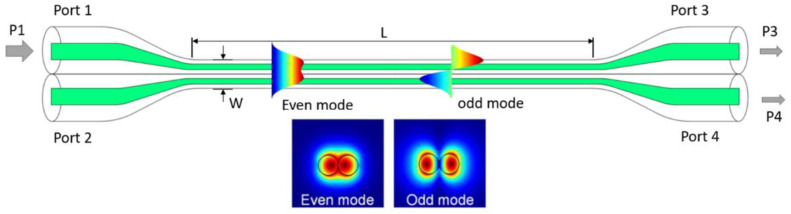
Typical structure of microfiber coupler.

**Figure 2:**
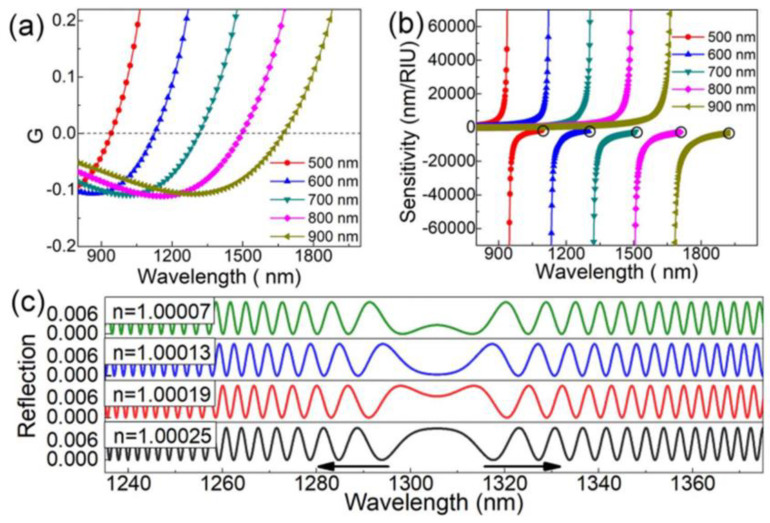
(**a**) Group-effective RI difference versus wavelength for microfiber couplers. (**b**) Calculated sensitivities as a function of wavelength. (**c**) Simulated reflective spectra with different surrounding RIs [13].

A typical microfiber coupler consists of two input ports, two output ports and a uniform waist region where two microfibers are closely contacted. The typical structure of a microfiber coupler is plotted in Figure 1. Light in single polarization is injected into input port 1, and odd and even surpermodes can be excited due to supermode theory. Then, power exchange occurs between the two modes as they propagate along the waveguide. The normalized output power at port 3 and port 4 can be expressed as

$$P_{3}=P_{1}cos^{2}\left(\frac{\pi L\Delta n_{eff}}{\lambda_{N}}\right)$$

$$P_{4}=P_{1}sin^{2}\left(\frac{\pi L\Delta n_{eff}}{\lambda_{N}}\right)$$

where $P_{1}$ is the input power of port 1, $\Delta n_{eff}$ is the difference between the even supermode and the odd supermode, L denotes the coupling length, and $\lambda_{N}$ is the probing wavelength. When sensing parameters (such as RI, strain, and temperature) change, the coupling process is influenced, so the output spectrum changes. By monitoring the probing wavelength shifts or the intensity fluctuations, the variation of surrounding physical/chemistry parameters can be estimated. Theoretically, the sensitivity of the giving probing wavelength $\lambda_{N}$ can be deduced as [14]

$$S=\frac{\partial \lambda_{N}}{\partial \delta }=\frac{\lambda_{N}}{\Delta n_{eff}-\lambda_{N}\cdot (\partial \Delta n_{eff}/\partial \lambda_{N})}\cdot \frac{\partial \Delta n_{eff}}{\partial \delta }=\frac{\lambda_{N}}{G}\cdot \frac{\partial \Delta n_{eff}}{\partial \delta }$$

where δ is the physical/chemistry parameter to be measured and $G=\Delta n_{eff}-\lambda_{N}\cdot (\partial \Delta n_{eff}/\partial \lambda_{N})$ is the group-effective RI difference between the even and odd surpermodes. When G approaches zero, the sensitivity significantly increases and can theoretically reach infinity. The DTP is defined where G=0 in Figure 2a. The sensitivity can be calculated, and the result is presented in Figure 2b. It can be found that the interference dips on left side of the DTP exhibit a positive sensitivity while those on the other side exhibit a negative sensitivity, which is consistent with the spectrum evolutions shown in Figure 2c. The dips of both sides drift toward the DTP with the increase in the surrounding RI. All the dips present linear responses towards the tiny RI variations. By tracing the dips and calculating the wavelength variations, the surrounding RI changes can be calculated. Theoretically, the sensitivity can reach infinity. Notably, due to the spectral width of the interferometric dip, the RI sensitivity cannot reach infinity. The excellent RI sensitivity properties provide great potential in the field of chemical and biochemical sensing. 

Guided by the DTP principle, several ultrasensitive fiber coupler-based sensors working near the DTP have been described. Li et al. presented a systematic study on the sensing performance of microfiber couplers at the DTP. They established ultrasensitive Y coupler sensors by omitting one of the input ports of a two-by-two coupler [13,16,17,25]. They proposed a refractometer that can sense subtle surrounding RI variations, with a maximum sensitivity of 59,624 nm/refractive index units (RIU) [16]. By sealing the same coupler sample in polydimethylsiloxane (PDMS), they built an ultrasensitive temperature sensor with a sensitivity of 16.78 nm/°C. Considering that an in-line structure may limit the application in narrow spaces, they proposed the use of sensing microprobe and realized gas refractive index sensing [13]. To further enhance sensitivity, a birefringence-induced Vernier effect in microfiber couplers was reported, and a higher sensitivity of 35,823.3 nm/RIU was achieved [17]. Then, this sensor was applied to detect human cardiac troponin, and a limit of detection of 1 ng/mL was achieved. Nevertheless, the RI sensing range was limited and needed to be expanded for application in more scenarios. Wan et al. fabricated a highly sensitive asymmetric microfiber coupler by heating and melting a section of SMF and a section of FMF together, and they developed RI and thermal sensing using only one sensor [26]. The well-designed asymmetric microfiber coupler allows for a high RI sensitivity of 10,662.4 nm/RIU within a large refractive index sensing range of 1.31–1.35. In addition to the applications mentioned above, a coupler-based sensor at the DTP can be used in other scenarios to sense physical parameters. Wen et al. manufactured a micro-coupler operating at the DTP that achieved an axial strain sensitivity of 166.9 pm/με and a linear range of 0–400 με, thus providing a new method for axial strain detection [27]. Furthermore, an ultrasensitive broadband acoustic sensor based on a micro-coupler attached to a diaphragm was reported. The micro-coupler sensor was sensitive to axial strain. It operated as a transducing element to monitor the deformation of a diaphragm driven by acoustic waves, achieving accurate measurements in the broadband acoustic wave range of 30~20,000 Hz with good linearity [28]. On the other hand, by coating a functional material onto the fiber surface, various novel sensing functions can be achieved. Pu et al. reported an ultrasensitive magnetic field sensor based on a magnetic fluid-coated microfiber coupler [29]. They reported a magnetic field sensitivity on the order of 10^2^ nm/mT, which was nearly two orders of magnitude higher than those of the previous studies. 

### 2.2. Fiber Grating

**Figure 3:**
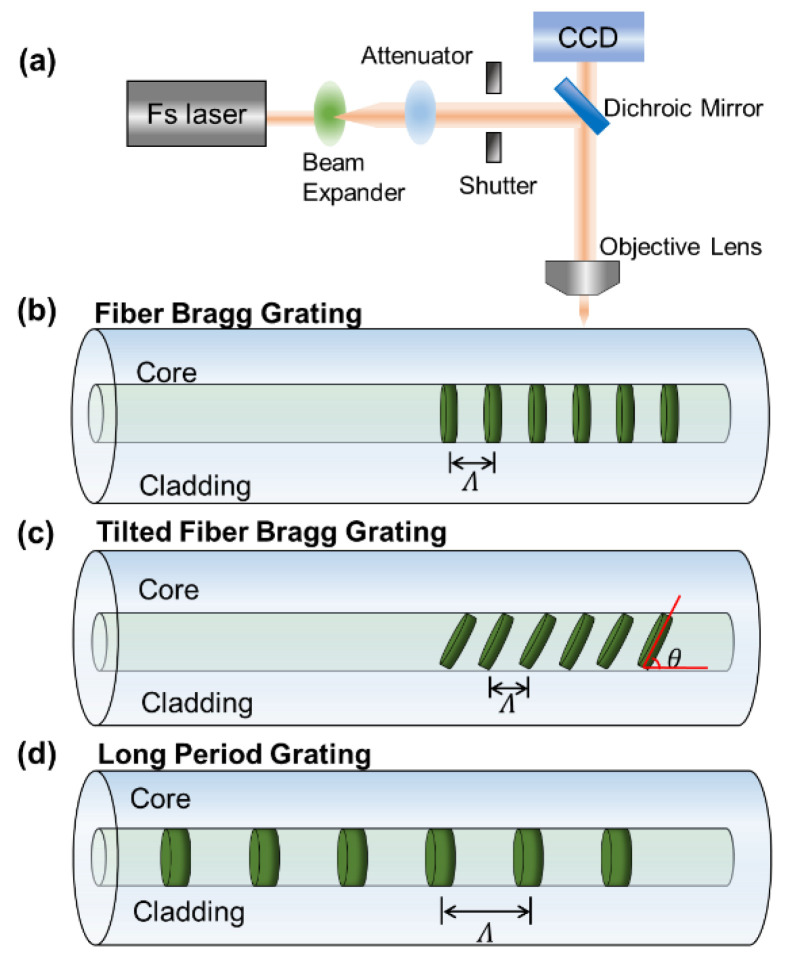
(**a**) Illustrative diagram of femtosecond laser processing. Schematics of the femtosecond-laser-induced grating and transmission principles: (**b**) FBG, (**c**) TFBG, and (**d**) LPG.

Fiber gratings consist of a periodic RI modulation along the core or the cladding of an optical fiber, allowing for the generation of resonances in the transmission or reflection spectrum by coupling light from the core mode to another co-propagating or back-propagating mode [19]. According to the grating period length, the fiber grating can be divided into two groups, short-period fiber grating (namely, fiber Bragg grating (FBG) and tilted fiber Bragg grating (TFBG)) and long-period grating (LPG), as shown in Figure 3. FBGs and TFBGs have a period of several microns, and LPGs have tens of hundreds of microns. The light-guiding mechanisms of them are different. FBGs can be seen as a stack of Fabry–Perot (FP) cavities that result in strong Bragg resonance through multi-cavity FP interference. The forward core mode is coupled with the back-propagation core mode at a specific wavelength. TFBGs are obtained by tilting FBGs at a certain angle. The forward core mode is coupled to back-propagation cladding modes at a specific wavelength [30]. The sensing performance of LPGs, FBGs and TFBGs is comparable. However, the former is easiest to prepare, mainly due to its longer period. Additionally, for LPGs, which have more modal energy in the form of the cladding mode (which is more sensitive to the surrounding environment), the light–matter interactions can be greatly enhanced and higher RI sensitivities can thereby be achieved. Researchers have proposed various LPG-based sensors with excellent sensing performance. Among the reported schemes, LPG sensors based on the DTP have been investigated and served as promising candidates for various RI sensing applications due to their superior sensitivity. Here, we briefly review LPG sensors based on the DTP. 

A LPG is characterized by a series of periodic RI changes in the order of hundreds of micrometers in the core of a SMF, as well as the wavelength-dependent coupling that occurs between the propagating core mode and the cladding modes. The relationship between the grating period and the resonance wavelength is described by phase-matching curves (PMCs), and the resonance wavelength $\lambda_{res}$ is represented as

$$\lambda_{res}=\left(n_{eff}^{co}-n_{eff}^{cl\;0,m}\right)\Lambda$$

where $n_{eff}^{co}$ and $n_{eff}^{cl\;0,m}$ are the effective RI of the fundamental core mode and the $m_{th}$ order cladding mode, respectively, and Λ is the period of the grating. For the specific cladding mode, the slope of PMCs exhibits a change in sign from positive to negative. Thus, for a given grating period, the transmission spectrum of the LPG exhibits two attenuation bands characterized by two resonance wavelengths. The DTP, also known as the turning around point, is the point in the PMC where the two resonant bands of the same cladding mode merge into a broader resonant band [19,31,32]. The DTP determines the condition of maximum sensitivity for each cladding mode. Therefore, a LPG can be designed to exhibit a very high sensitivity for a particular wavelength by selecting a cladding mode and period at, or very close to, the DTP. Researchers have proved that a high sensitivity can be achieved around the DTP. The resonant wavelengths on the opposite sides of the DTP show different wavelength drift tendencies with physical or chemical perturbations, which are consistent with those of the coupler-based sensors at the DTP. Therefore, some authors have used the wavelength separation between both resonance dips to measure sensitivity, which can double the value obtained with a single resonance.

For a LPG, the DTP can be reached by three methods: wet etching the fiber cladding, adjusting the grating periods, and using function material-coating [19,32,33]. As one of the most fundamental physical parameter sensing methods, RI measurement with high accuracy and precision is increasingly finding applications in environmental monitoring, biochemistry, and medical diagnosis. Regarding refractometers, Zhang et al. reported on a DTP-based LPG fabricated in SMF at a 2 μm waveband [34]. Through a high sensitivity of 8233.3 nm/RIU was achieved, the optical sources and detectors were more expensive than that of shorter wavelengths. Wang et al. reported an ultrasensitive refractometer based on a helical long-period fiber grating operating near 1.5 μm, which is a typical optical communication band [35]. They also proved that the proposed sensor structure had the characteristics of a low temperature and strain crosstalk. In addition to RI sensing, LPGs can detect other physical parameters. Villar et al. proved that sensitivities up to 20 pm/με could be attained using DTP-based LPGs fabricated via the wet-etching method [36]. They demonstrated that the sensitivity in the grating region did not depend on the order of the cladding mode responsible for the attenuation band but on the proximity to the DTP for each mode order. However, the wet-etching process may lead to the increased fragility of a sensor. Except for inscribing SMFs to obtain a LPG, Zhang et al. proposed a novel LPG prepared by multimode fibers and developed a sensitivity-enhanced strain sensor with a sensitivity of 15.06 pm/με [37]. 

**Figure 4:**
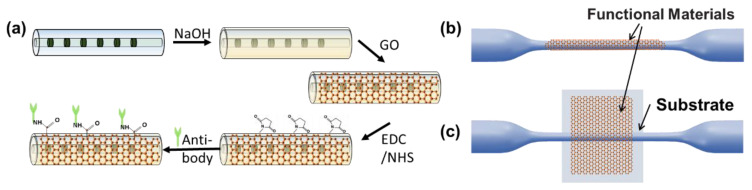
(**a**) Schematic illustration of the fabrication of a graphene oxide-coated LPG immunosensor. (**b**,**c**) Functional material integration into the optical fiber waveguide platform.

On the other hand, optical fiber sensors with novel sensing functions, multi-parameter sensing ability, and high efficiency are important for practice applications. With the assistance of function material-coating, researchers can create innovative LPG-based sensors working near the DTP. Mateusz et al. proposed an Al_2_O_3_-nanocoated LPG, the temperature sensitivity of which was increased to 2.5 times higher than that of uncoated samples because of the high thermo-optic coefficient of the Al_2_O_3_ nanocoating material [33]. Researchers expect to find another coating material whose thermo-optic coefficient compensates for the intrinsic temperature sensitivity of the LPG. As mentioned above, LPGs offer extraordinary sensitivity to changes in the external RI, which make them promising for label-free biosensing. A highly sensitive LPG label-free immunosensor was fabricated to detect T7 bacteriophages [38]. The fiber surface was functioned by 3-(triethoxysilyl) propylsuccinic anhydride to covalently bind anti-T7 antibodies. In this way, the T7 phage could be effectively detected. Similarly, Wu et al. designed and prepared a graphene oxide-coated LPG immunosensor that showed a high specificity to the H5N1 virus [39]. Quero et al. reported a fiber optic nano-optrode based on LPGs for the detection of human thyroglobulin (TG). The LPG-based biosensor was coated with a single layer of atactic polystyrene (aPS) onto which a specific, high-affinity anti-Tg antibody was adsorbed. In this way, the proposed LPGs could realize the label-free detection of Tg in the needle washouts of fine-needle aspiration biopsies [40,41]. Their group additionally proposed an automated optical assay based on label-free optical fiber optrodes for the fast detection of class C β-lactamases (AmpC BLs). Reflection-type LPGs with double-layer (aPS and poly(methylmethacrylate)-co-methacrylic acid (PMMA-co-MA)) deposition on the surface have been used as highly sensitive label-free optrodes. The automated system tested in AmpC solutions at increasing concentrations demonstrated a limit of detection (LOD) of 6 nM. The proposed optical assay-based LPGs showed great potential for applications in precision biomedicine detection [42]. Additionally, the electrochemical processes with integrated optoelectrochemical functional materials can be investigated [43]. We illustrate the typical functional material integration of a DTP-based fiber platform in Figure 4a,b. Generally, the sensitivity of a LPG is relatively low due to the limited evanescent field, and the fabrication process requires a femtosecond or CO_2_ laser, which is of high cost. Therefore, it is vital to develop a sensor based on the DTP with a lower cost and higher sensitivity. 

### 2.3. In-Fiber Interferometer

**Figure 5:**
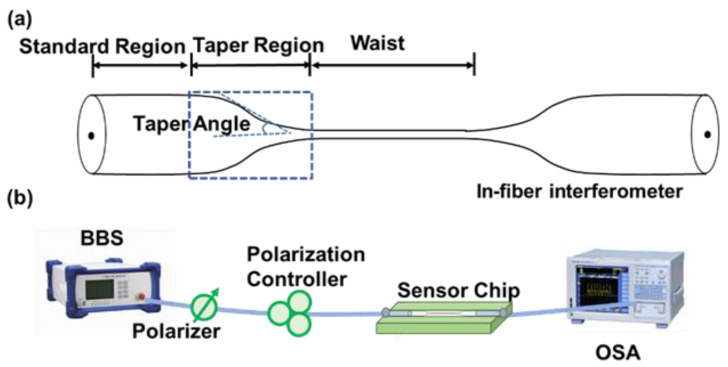
(**a**) Schematic of in-fiber interferometric structure. (**b**) Typical experimental setup of an in-fiber interferometer.

Researchers have shown great interest in in-fiber interferometer-based sensors because of their more flexible structures and easier fabrication compared with those of the configurations mentioned above. In-fiber Mach–Zehnder microfiber interferometers (MZIs) are broadly applied for optical sensing due to their simple structures and compact sizes [44,45,46]. A typical microfiber structure comprises standard regions, taper regions, and a segment of the tapered fiber, as illustrated in Figure 5a. It should be mentioned that a taper profile can be divided into adiabatic or non-adiabatic tapers, which influences mode excitation and selection [47,48,49]. To evaluate the influence of the taper profile, many mathematical models have been used to discuss the taper shape, such as the linear, raised cosine, and modified exponential taper models [50]. Additionally, taper length has a direct effect on the adiabaticity of a taper. It is necessary for the taper length to not exceed the minimum taper length that guarantees that the fundamental mode is adiabatic along the entire length of the taper [9,49]. The DTP has been experimentally observed and demonstrated in different in-fiber MZIs based on multi-mode or polarimetric interference. 

The sensing mechanism of in-fiber MZIs is mainly based on the interference of multiple modes. The incoming mode is split into two different modes due to mode field mismatch. These two co-propagating modes accumulate a phase difference after propagating along the fiber length, and then interference occurs when the two modes are combined. The intensity of the transmission spectrum can be expressed as

$$I=I_{1}+I_{2}+2\sqrt{I_{1}\cdot I_{2}}\cos\left(\varphi \right)$$

where $I_{1}$ and $I_{2}$ are the intensities of the two co-propagation modes and φ is the difference of the propagation phase, which may emerge from different paths (birefringence or modal dispersion). The typical experimental setup of an optical microfiber sensor is illustrated in Figure 5b. A broadband light source (BBS) is employed as the incident light, and an optical spectrum analyzer (OSA) is used to record the output spectra. In this review, we divide intermodal MZIs into two parts according to the fiber employed as the sensing unit: standard fiber-based interferometers and specialty fiber-based interferometers. Standard fibers denote commercially available fibers, such as SMFs and few-mode fibers (FMFs). Specialty fibers are specifically designed fibers. 

#### 2.3.1. Standard Fiber-Based Interferometers

The mode interference in DTP in-fiber MZIs mainly occurs between the fundamental mode and the well-selected high-order mode or between two orthogonal polarization modes. Compared with polarimetric interference, where the polarization-dependent modes couple with others utilizing a unique detection system, in-fiber MZIs based on multimodal interference are more stable, compact, and polarization-independent.

Researchers have proposed a series of standard fiber-based in-line interferometers with flexible and compact configurations. Lu et al. spliced a section of FMF between two sections of SMFs and obtained a sandwich structure [51]. A DTP, also called critical wavelength (CWL), appeared in the transmission spectra of the multimode interference [15]. Simultaneous measurements of strain and temperature were carried out. However, the temperature and strain sensitivities of 0.042 nm/°C and −0.001 nm/µε, respectively, were relatively low, which was not competitive with other fiber sensors. On the other hand, by tapering the standard fibers to the micro size and controlling the transition segments to meet the non-adiabatic conditions, a higher-order mode could be excited and effectively selected. Wang et al. fabricated a tapered SMF modal interferometer, and an RI sensitivity over 126,000 nm/RIU was obtained, allowing for the detection of sodium nitrate in an aqueous solution [52]. Additionally, Wei et al. comprehensively analyzed the influence of the taper length and waist length of tapered SMFs on the DTP and the evolution of the spectra, offering guidance for the design and preparation of SMF-based interferometers [53]. Furthermore, Guan et al. numerically simulated and experimentally revealed that two DTPs formed with decreases in the microfiber waist diameter, refining the DTP sensing theory [54]. 

Though typical tapered SMF-based microfibers have simpler configurations and lower costs than other fiber devices, their transition segments must be abrupt to meet the non-adiabatic requirement and therefore effectively excite higher-order modes, which significantly increases the difficulty of preparation. Zhou et al. proposed an effective method to ease the difficulty of mode excitation. They developed a zigzag-shaped microfiber and found that a portion of the fundamental mode was effectively transferred into higher-order modes [20]. By carefully controlling the bending angle of the zigzag shape, high-contrast interference fringes of the coupling modes can be formed, dramatically improving the RI sensing performance. However, the transmission-type structure may induce inconvenience in practice, particularly in semi-enclosed or narrow spaces. Sun et al. fabricated a reflective microfiber modal interferometer that assisted with the Fresnel reflection at the end of microfibers, realizing the real-time monitoring of target DNA [10]. Then, the researchers coated the SMF surface with functional materials to achieve a better sensing performance or special sensing functions. Zhou et al. modified a tapered SMF surface with thin polydimethylsiloxane (PDMS) film and obtained an ultra-sensitive gas pressure sensor [55]. Shen et al. created a high-temperature sensor using ZnO composite graphene-coated fibers [56]. 

However, for all the reported in-line standard fiber-based interferometers working near the DTP, the operation bandwidths of the probing wavelength for high-sensitivity regions are narrow. As a result, the twin dip next to the DTP is hard to experimentally observe. Only positive sensitivities can be obtained, which increases the experimental complexity and decreases the detection efficiency. Additionally, considering the spectral width of the interferometric dips, a broader operation band can afford a route for a liberal waist-length condition, a compact structure, and a high measurement efficiency. On the other hand, the excitation of a high-order mode depends on the taper parameter during the fabrication process, which influences the interferometric visibility and the detection accuracy. More specifically, the interferometric visibility of the transmission spectrum depends on the similarity between the coupling modes [9]. Ideally, the intensity ratio of the interfering modes approximately approaches 1 (the input power is equally coupled to both modes). The visibility is degraded when there is any dissimilarity between the coupling modes. In practice, modes of different amplitude are excited in the tapering region, and the transmission loss of each mode is generally diverse, depending on the condition of excitation. Therefore, microfiber structures should be strictly designed and manufactured due to the limitations of the operation bandwidth and interferometric visibility, which significantly decreases fabrication reproducibility. However, the challenges facing broadband sensing properties and high spectral extinction ratios remain unexplored.

#### 2.3.2. Specialty Fiber-Based Interferometers

Researchers have proposed mode dispersion theory to overcome challenges regarding high-sensitivity limited operation bandwidths and low fabrication tolerances, and specialty optical fibers with specifically tailored parameters have been employed as sensing units. To improve measurement accuracy and ease the strict requirements on taper parameters, Guan et al. fabricated highly birefringence elliptic/rectangular microfibers with a CO_2_ laser, and they obtained an ultrasensitive sensor with high spectral extinction ratios in Sagnac loops [11,57,58]. As mentioned above, the extinction ratio is correspondingly degraded when there is any dissimilarity between two modes. For the reported standard fiber-based interferometers, controlling the higher-order mode excitation and power distribution of modes remains challenging, as it requires the strict control of taper parameters, such as drawing velocity and duration, during fabrication [48,49,50]. Elliptic microfibers rely on Sagnac-based polarimetric interference between two modes of orthogonal polarization with similar mode field distributions, which greatly improves the extinction ratios. However, the fabrication of elliptic microfibers requires a special CO_2_ laser machining system to precisely control the microfiber cross-sections, which greatly increases the fabrication cost and decreases the devices’ repeatability. Additionally, the importance and theoretical analysis of high-sensitivity operation bandwidths remain to be resolved. 

**Figure 6:**
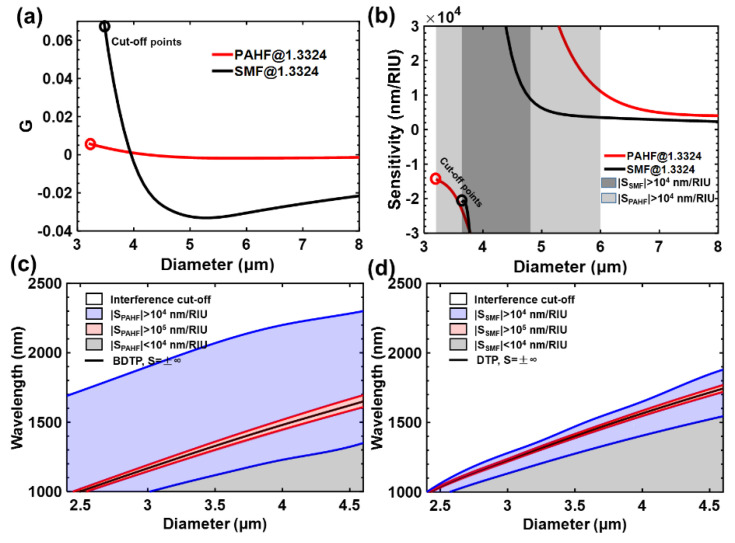
(**a**) Group birefringence G and (**b**) calculated sensitivity for PAHF and SMF microfibers with different diameters. (**c**,**d**) RI sensitivity and interference cutoff regions of PAHF and SMF microfibers, respectively.

Our group proposed a mode dispersion engineering method to broaden the operation bandwidth and enhance the fabrication tolerance of sensors [9,21]. Specialty fibers with expected mode dispersion properties were obtained by designing and modifying the material index. In this way, an ultrasensitive broadband sensor with high spectral extinction ratios utilizing the polarimetric interference of a tapered PANDA-air-hole fiber (PAHF) was proposed [9]. The proposed PAHF was specifically designed by introducing double air holes into the cladding, and we obtained both high birefringence and unique group birefringence. More specifically, the material index of the PAHF was flexibly engineered by designing the internal microstructures, so the mode dispersion properties could be optimized and manipulated. In this way, the diameter/wavelength dependence of the group birefringence G was significantly reduced, as shown in Figure 6a. Furthermore, the workable diameter range for realizing an ultrahigh sensitivity was twice as large as standard tapered fiber-based interferometers. The calculated results are illustrated in Figure 6b. The high-sensitivity regions for PAHF and SMF-based microfibers of the same diameter range are plotted in Figure 6c,d. The broadband operation of PAHF was achieved, and the efficiency of the dispersion engineering theory was proved. We further proposed an in-line multimodal interferometer employing a well-designed single-stress fiber [21] that exhibited the advantages of a large operating bandwidth with a more compact structure compared with a PAHF-based interferometer.

The microstructures of specialty fibers affect their transmission characteristics and, thus, their sensing performance [59,60]. There are many design degrees of freedom with various optimization parameters, so researchers have proposed many fiber design strategies. The principle of the traditional method is to obtain a desired fiber microstructure through parameter scanning. This method mainly relies on intuition to search for the optimum design scheme in a large number of samples. Fiber microstructures are randomly designed without following specific design concepts, so such methods are generally time-consuming, especially for multi-parameter structures that need fine meshes for high precision. Various algorithms have been utilized to realize efficient and workable designs that can be transferred to specialty fiber design [61,62]. 

Automatic advanced fiber design methods can be further divided into forward design and inverse design [63]. For forward design, a designer conceives of a fixed fiber structure, such as the material index and geometry of the structure, as a starting point of the design. By scanning the microstructure parameters with advanced algorithms, the expected specialty fiber can be well-designed. Researchers have used the genetic algorithm (GA) and particle swarm optimization (PSO) methods in fiber-microstructure designs [62,64,65]. Cao et al. designed the dispersion of a multi-loop low-mode fiber based on GA and realized the zero-dispersion optimization of four modes [66]. However, this forward process is labor-intensive and may exclude a wide range of design results, some of which may have more potential than traditional structures. On the other hand, inverse design allows designers to directly specify performance targets and manufacture constraints, simply by inputting a set of desired sensing characteristics and then using an optimization algorithm to generate a predicted solution. In principle, inverse-design methods can explore the full space of specialty fibers. For example, Du et al. developed a machine learning method using a neural network to inversely design a desired FMF based on a multiple-ring structure, providing high accuracy, high efficiency, and low complexity for the fast and reusable design of optical fibers [67]. 

**Figure 7:**
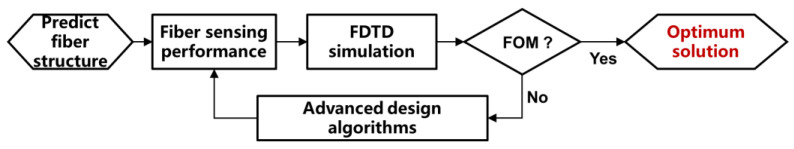
Schematic diagram of the advanced design of optical fiber microstructures.

To obtain a specialty fiber-based interferometer near the DTP with a large operation bandwidth and an enhanced fabrication tolerance, it is necessary to carefully design fiber microstructures to meet two conditions. The specific design process is presented in Figure 7. Firstly, within the given operation bandwidth, the DTP needs to be implemented and observed; that is, there is a point where the group-effective RI difference is equal to zero. Thus, ultrasensitive microfibers can be theoretically created. Secondly, the diameter/wavelength sensitivity of G needs to be reduced; that is, dG/dD or dG/dλ (where D is the diameter and λ is the probing wavelength) needs to be modified to numerically approach zero. Afterward, these two requirements need to be input into the fiber design model, and with the help of advanced algorithms, the optimal specialty fiber structure can be obtained. More promisingly, advanced design methods can be used to develop various specialty fiber-based sensors with high performance and great potential for instantaneous applications in the optical fiber industry.

## 3. Challenges and Opportunities

Over the past fifteen years, researchers have made a significant progress in the field of ultrasensitive fiber sensing based on DTP theory. We present different optical structures in Table 1 so that readers can compare the sensing performance of different optical platforms. 

**Figure 8:**
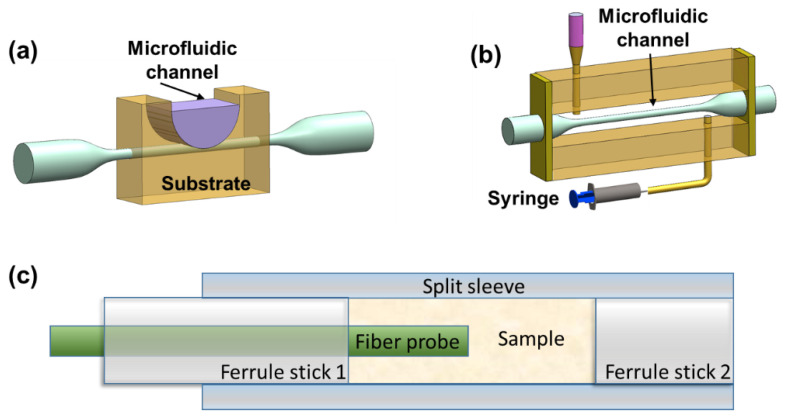
Schematic diagrams of fiber sensor package. (**a**,**b**) optical fiber integration to microfluidic channel for sensing; (**c**) optical fiber probe for sensing.

To date, many types of DTP-based fiber sensors have been employed for physical, chemical, and biological applications, showing great functional scalability. However, few of them have migrated to the commercialized sensor level, and there is still a long way to go from the laboratory to actual use. First, the repeatability of fabrication, stability of use, and limitation of sensing range remains serious challenges. For example, fibers with thin waists are fragile and sensitive to surrounding disturbances. Micro-bending may excite undesired modes, degrading measurement accuracy. Although DTP-based fiber sensors can provide advantages over traditional fiber sensors in terms of ultrahigh sensitivity and compact size, other sensing platforms such as micro-electro mechanical systems (MEMS) [70] and integrated photonic silica-based devices [71] are more competitive and have a more mature industrial technology base. Nevertheless, researchers have made much progress in addressing the emerging problems and improve the sensing performance of DTP-based sensors. Several approaches have been proposed to solve the first problem mentioned above. The effectiveness of the mode dispersion method has been theoretically demonstrated, experimentally proven, and combined with automatic fabrication and online monitoring systems [10,16,21]; production repeatability can be further improved. Additionally, researchers have conducted a series of studies on the packaging of fiber sensors, as shown in Figure 8 [10,16,72]. For example, a microfiber was embedded in low-RI materials and integrated with well-design microfluidic chips [1,6,73]. However, there are some critical technical problems that need to be solved. Firstly, an automatic design–fabrication–packaging system with acceptable cost has not been established, which is of great significance to the further development of DTP-based fiber sensors. Furthermore, the advantages and unique qualities of DTP-based fiber sensors compared with different types of sensing technology, such as ultra-compactness, mechanical flexibility, biocompatibility, and electromagnetic interference resistance, need to be clarified. We think that attention can be focused on applications of label-free biological sensing, the study of coating functionalization materials, the enhancement of sensing sensitivity, and demodulation algorithms of multi-parameter sensing [5,8,25,74,75]. The concept of “lab-on-fiber” also needs to be highlighted [76]. Lab-on-fiber technology integrates the advantages of optical fiber optics, materials science, biomedicine, and other disciplines [72]. Combined with lab-on-fiber technology, a plug-and-play remote microsensor suitable for limited environments can be developed, which would have great significance and potential value in the field of multi-parameter micro-space sensing, especially for in vivo biosensing. Undoubtedly, the development of DTP-based fiber sensing platforms will proceed to generate new opportunities for their use in practical applications. 

## 4. Conclusions

In this paper, we comprehensively reviewed the state-of-the-art ultrasensitive microfiber sensors based on DTP technology. DTP sensing mechanisms have been introduced, and sensitivity can be significantly improved by taking full advantage of DTP theory. DTP-based sensors have developed into three different types, and recent advances and applications have been presented. We also evaluated each sensing scheme. Finally, we discussed the challenges and opportunities of DTP-based sensors. We believe that DTP-based sensors will continue to develop and become useful in various industries and commercial applications.

## Figures and Tables

**Table 1:** Fiber sensor performance based on the theory of the dispersion turning point.

Configuration	Sensing Parameter	Surface Functionalization	Sensitivity/Limit of Detection	Diameter	References
fiber coupler	Temperature	Sealing in PDMS	16.78 nm/°C	3 μm	[16]
	Axial strain	-	166.9 pm/με	2.53 μm	[28]
	Acoustic wave	-	1923 mV/Pa	1.6 μm	[68]
	gas RI	-	92,020 nm/RIU	1.4 μm	[13]
	RI	-	35,823.3 nm/RIU	3.2 μm	[17]
	Tumor biomarkers	Immobilized with antibody	34.6 fg/mL	~2.8 μm	[26]
LPG	RI	-	25,546 nm/RIU	121 μm	[35]
	Temperature	Coated with Al_2_O_3_	8200 nm/RIU	125 μm	[33]
	RI	Coated with TiO_2_	8051.4 nm/RIU	192.5 μm	[69]
	Class C β-lactamases	Coated with aPS and PMMA-co-MA	6 nM	-	[42]
	Thyroglobulin	Coated with aPS	<6 pM	-	[41]
	H5N1 virus	Coated with graphene oxide	1.05 ng/mL	<125 μm	[40]
Standard fiber-based interferometer	Nitrate	-	126,000 nm/RIU	3.0 μm	[45]
	Gas RI	-	-69,984.3 nm/RIU	~2 μm	[53]
	RI	-	1.46 × 10^5^ nm/RIU	2.3 μm	[20]
	Gas pressure	-	0.295 nm/KPa	~1.57 µm	[11]
	DNA	Immobilized with single-stranded DNA probes	0.03 nm/ pM	6.6 μm	[10]
Specialty fiber-based interferometers	RI	-	47,223 nm/RIU	3.2 µm	[9]
	RI	-	30,563 nm/RIU	2.3 µm	[21]

## Data Availability

Not applicable.
